# The developmental process of suicidal ideation among adolescents: social and psychological impact from a nation-wide survey

**DOI:** 10.1038/s41598-023-48201-6

**Published:** 2023-11-28

**Authors:** Antonio Tintori, Maurizio Pompili, Giulia Ciancimino, Gianni Corsetti, Loredana Cerbara

**Affiliations:** 1 Institute for Research on Population and Social Policies, National Research Council of Italy, 00185 Rome, Italy; 2 Department of Psychiatry, Sant’Andrea Hospital, Sapienza University of Rome, 00189 Rome, Italy; 3 Italian National Institute of Statistics, 00184 Rome, Italy

**Keywords:** Psychology, Risk factors

## Abstract

Suicidal ideation is a multifactorial phenomenon that is increasingly prevalent among adolescents, especially following the impact of Covid 19 pandemic on their mental health. Its analysis necessitates an interdisciplinary approach that simultaneously considers sociological and psychological perspectives, especially looking at the role of interpersonal relationships and structural inequalities. The present study, based on a face-to-face survey conducted with a representative sample of 4288 adolescents, aims to identify the factors that most differentiate individuals with and without suicidal ideation, proposing a descriptive model of development process of this phenomenon with reference to the Italian context. We analysed variables related to socio-demographic status, relational status, social interactions, and psychological well-being using multiple correspondence analysis and logistic regression models. The results provide evidence for the existence of a direct association between negative psychological status and suicidal ideation but clarify that the psychological aspects are associated with sociodemographic characteristics and have their origins in the social sphere.

## Introduction

Suicide, which is defined as "death caused by self-directed injurious behavior with intent to die as a result of the behavior"^1^, represents a major global health issue. According to the World Health Organization (WHO), over 700,000 individuals die by suicide each year, with approximately twenty attempted suicides for every completed one, and even more individuals experiencing suicidal ideation^2^. In 2019, 77% of suicides occurred in low-and middle-income countries, while high-income countries recorded the highest age-standardized suicide rate of 10.9 per 100,000 individuals. Moreover, the suicide rate for men (12.6) was more than double that of women (5.7), and this gender disparity further widens in wealthier nations^3^, despite women have higher rates of suicidal ideation, suicide attempts, and non-suicidal self-harm^4^. According to the most recent data, at a global level suicide is the third leading cause of death among girls aged 15 to 19 years and the fourth leading cause among boys of the same age group^3^. Furthermore, adolescents represent a social group where suicidal ideation is more widespread^5^. In Italy, where 3748 people died by suicide in 2020 (78.5% men), the suicide mortality rate remains relatively low at 5.61 compared to the European average of 10.24, showing a slight decrease compared to the average of the previous five-year period from 2015 to 2019, with a decline of 4%^6^.

Suicide is a complex phenomenon to study, mainly due to the difficulty in retrospectively analysing suicide cases^7^. This is why scientific research often studies suicidal ideation and behaviour, since it is possible to collect data directly on the subjects involved in the phenomenon. However, data on these topics can be inconsistent due to the different operational definitions and measurement approaches which have been employed^8^. Additionally, cultural differences and the stigma surrounding suicide can further limit reporting suicidal ideation and behavior, making cross-cultural comparative studies less reliable^7^.

Suicidal ideation, also known as thoughts about planning to commit suicide, includes contemplations, wishes, and preoccupations with death^8^.

The object of the present study is suicidal ideation among adolescents, due to the fact that the adolescence is notoriously an evolutionary phase that has substantial importance in the life of the individual, as it represents the transition to adulthood^9^. The synthesis of all the experiences lived in the family, at school and in society can in fact favor or inhibit the full inclusion of a subject in the social context, affecting the level of well-being.

This phenomenon has significantly increased worldwide following the severe impact of the COVID-19 pandemic on adolescents' mental health, as reported by numerous studies published over the last three years^10^. The sudden disruption of face-to-face relationships and the consequent shift to virtual interactions have had profound repercussions on the well-being of young people, leading to exponential growth in anxiety, depression, and other primary negative emotions^11,12^. Many studies have also revealed increased suicidal ideation during the COVID-19 pandemic due to social isolation and loneliness^13^. Their results are consistent with what has been recorded by healthcare organizations. In 2020, mental health-related emergency department (ED) visits among youth aged 12–17 in the US increased by 31% compared to 2019. Moreover, between 21 February and 20 March 2021, ED visits for suspected suicide attempts were 50.6% higher among girls aged 12–17 years and 3.7% higher among their male peers than in the same period in 2019^14^. Additionally, a study on suicide prevention telephone helplines, which collected data on 8 million calls from 19 countries, found that call volumes peaked six weeks after the initial COVID-19 outbreak at 35% above pre-pandemic levels due to widespread feelings of loneliness and fear^15^.

Furthermore, data from the Italian association providing emergency and telephone support services indicate that in 2021, requests for help from people considering suicide or worried about the possible suicide of someone close increased by 55% compared to 2020 and nearly quadrupled compared to 2019, particularly among young people^16^.

It is important to emphasize that suicidal ideation does not automatically lead to suicidal action. According to some pre-pandemic studies, only one-third of individuals who have experienced suicidal ideation attempt suicide^17,18^. Based on this assumption, theories within the ideation-to-action framework seek to explain the transition from suicidal ideation to suicide attempts by differentiating the factors involved in each process^19^. Within this theoretical framework, the interpersonal theory of suicidal behavior highlights the importance of social interactions in developing suicidal ideation^20–22^.

Nonetheless, this popular theory that explains suicide through the simultaneous presence of only three risk factors—acquired capability for suicide, thwarted belongingness, and perceived burdensomeness—appears to oversimplify the multifaceted and contextual nature of the phenomenon^23^. Although it has long been recognised that suicide, as well as suicidal ideation, is a cultural construct that should be analysed in reference to norms and socio-cultural attitudes specific to each community^24^, the inclination to achieve a universal understanding of suicide tends to neglect the cultural underpinnings of deeper distress, resulting in reductionist and decontextualized theories^23^. This highlights the need of transcending the objectification of knowledge, which exposes notable deficiencies in universalist theories, through an interdisciplinary examination of the phenomenon, encompassing both quantitative and qualitative approaches that simultaneously engage sociological and psychological variables^25^ emphasising the relational^26^ and emotional factors without neglecting the role of interpersonal contexts of communication and structural social inequalities.

Currently, with specific reference to adolescents, higher rates of suicidal ideation have been associated with high levels of psychological distress^27^ and low self-esteem^28^. The presence and frequency of suicidal ideation during this delicate phase of life have been also associated with the following social factors: negative family climate and scarce quality of relationships with parents^29,30^ and friends^31,32^, pressure related to academic performance^33,34^, negative school environment^35^ but also bullying and cyberbullying victimization^36,37^ and drug and alcohol consumption^17^. However, in the implementation of preventive health interventions the weight of social factors has not been adequately considered^25^, especially concerning the extent of social integration and the complex interplay between relational dynamics and psychosocial factors in the development of suicidal ideation.

It is worth noting that until the 1800s, suicide was perceived as a strictly personal act, devoid of social and cultural considerations. It was only with Emile Durkheim's research^38^ that suicide began to be viewed as a "social fact" associated to social integration and community norms. However, achieving a comprehensive understanding of suicide risk as a multifactorial phenomenon remains challenging, given the array of variables involved and the roles of both distal and proximal factors in determining vulnerability to such risk within a specific cultural context^39^. However, when focusing on the phenomenology of the suicidal scenario, a straightforward construct referring to mental pain appears helpful in understanding the source of suffering for those experiencing suicidal wishes^40–42^. In an increasingly interconnected society, this suffering could have even deeper roots in the state of interpersonal interactions than in the past.

The aim of the present study is to identify the factors that differentiate individuals with and without suicidal ideation and the relationships among these factors, and propose an interdisciplinary, descriptive (non-predictive), and contextualized model for understanding the development process of suicidal ideation among adolescents in Italy. This study is based on a post-pandemic quantitative cross-sectional survey conducted with a representative sample of 4,288 Italian adolescents attending public upper secondary schools, using the CAPI (Computer Assisted Personal Interview) technique. To address the multidimensional nature of the suicide phenomenon, we simultaneously consider socio-demographic, psychological, and sociological factors to enhance the scientific understanding of the aetiology of suicidal ideation among adolescents. Indeed, the adoption of an interdisciplinary approach involves the consideration of a wide array of variables to better understand the phenomenon^43^.

More specifically, we included in the analysis four groups of variables: (1) variables related to the socio-demographic status (sex, type of school attended, class, geographical area, parental cultural and economic status, religious beliefs and citizenship); (2) variables related to the relational status (quality of friendship, friendship network size, friendship satisfaction and quality of parent–child relationships); (3) variables related to social interaction (hyperconnection, experiences with bullying and cyberbullying, sense of belonging to the school community, academic performance, body satisfaction and tolerance towards the use of alcohol and other substances); (4) psychological variables (psychological distress, self-esteem, attitude towards the future, happiness, satisfaction, primary emotions). Thus, the objective is to understand the weight of sociological, demographic and psychological factors in the adolescents’ suicidal ideation, i.e., to understand the interconnections and specific roles of the four groups of variables mentioned above. The study will also place specific emphasis on differences between girls and boys, as the risk of suicidal ideation displays significant variations by sex, which is a critical aspect when dealing with the adolescent population^18,44^.

To identify the factors that most differentiate individuals with and without suicidal ideation, and then theorise a descriptive model of development process of this phenomenon among Italian adolescents, the following hypotheses have been formulated:

- H1. Suicidal ideation varies based on socio-demographic factors.
- H2. Psychological variables have a direct impact on suicidal ideation.
- H3. Socio-demographic status, relational status and social interaction directly influence psychological variables.

## Results

Table 1 shows the variations in the frequency of suicidal ideation according to socio-demographic variables, including sex, geographical area, citizenship, class, type of school, religious beliefs, and parental cultural and economic status. It is important to note that our variable for suicidal ideation is ordinal and qualitative and participants were asked to rate the frequency of their suicidal thoughts on a five-point scale up to the time of the interview.

**Table 1:** Frequencies of suicidal ideation by socio-demographic variables (n).

	More than once	Once	Never
Sex			
Male	16.9% (425)	21.5% (541)	61.6% (1555)
Female	28.6% (505)	25.6% (453)	45.8% (809)
Total	21.7% (930)	23.2% (994)	55.1% (2364)
Geographical area			
South	18.1% (157)	22.3% (193)	59.6% (516)
Islands	21.9% (181)	21.2% (175)	56.9% (470)
Centre	22.8% (195)	22.8% (195)	54.4% (467)
North East	22.6% (195)	24.7% (213)	52.7% (456)
North West	23.1% (202)	24.9% (218)	52.0% (455)
Class			
I	20.3% (191)	23.4% (220)	56.3% (529)
II	23.4% (207)	22.7% (201)	53.9% (476)
III	20.9% (172)	24.5% (201)	54.6% (449)
IV	23.3% (196)	22.9% (193)	53.8% (452)
V	20.5% (164)	22.3% (179)	57.2% (458)
Citizenship			
Italian	21.0% (835)	23.1% (917)	55.9% (2223)
Foreign	30.4% (95)	24.6% (77)	45.0% (141)
Type of school			
Lyceum	23.0% (383)	24.0% (401)	53.0% (883)
Technical institute	18.7% (256)	22.6% (309)	58.7% (805)
Vocational school	23.3% (291)	22.7% (284)	54.0% (676)
Religious beliefs			
Non-believer	26.2% (517)	23.9% (470)	49.9% (985)
Believer	16.6% (307)	22.8% (422)	60.6% (1125)
Parental cultural status			
Low	23.1% (194)	20.7% (174)	56.2% (471)
Medium	22.1% (466)	23.0% (486)	54.9% (1157)
High	20.2% (270)	24.9% (334)	54.9% (735)
Parental economic status			
Low	28.3% (134)	27.7% (131)	44.0% (208)
Medium–low	23.1% (315)	22.3% (304)	54.6% (744)
Medium–high	19.9% (380)	22.2% (425)	57.9% (1107)
High	18.7% (101)	24.9% (134)	56.4% (304)

Overall, more than half of the respondents (55.1%) reported never having experienced suicidal thoughts, while 23.2% reported having these thoughts only once, and the remaining 21.7% reported experiencing them more than once. The prevalence of this phenomenon is consistently higher among girls, with 4 out of 10 girls reporting never having had suicidal thoughts, compared to 6 out of 10 boys. From a geographical perspective, the results show a higher frequency of suicidal thoughts among adolescents living in the country’s northern areas, with the lowest prevalence rate recorded among respondents from the south of Italy. There is no linear variation in suicidal ideation based on the class attended by the respondents. Regarding citizenship, foreign students exhibit higher rates of both the presence and frequency of suicidal ideation compared to their Italian peers. Moreover, technical school students had a lower rate of suicidal ideation than both lyceum and vocational school students. Suicidal ideation also varies according to religious beliefs, with lower rates observed among believers. Parental cultural status shows slight variations, with higher rates reported among students from low cultural backgrounds. Additionally, the frequency of suicidal ideation increases as parental economic status decreases.

Below, we present the findings from the multinomial logistic regression models aimed at identifying the developmental process of suicidal ideation among adolescents. Since most of the associations are similar for both male and female adolescents, we will primarily focus on the results of the overall model that includes all respondents. Nevertheless, it is important to note that separate models were always run for boys and girls, and the significant variations based on sex will be emphasise. Table 2 illustrates the significant results of the first multinomial logistic regression model, which identify the relations between socio-demographic characteristics, employed as independent variables, and the individual well-being of adolescents, the dependent variable. Based on the results, the respondents’ sex has not shown a significant relationship with the probability of having a negative individual status. However, this probability is 1.5 times higher for lyceum students compared to vocational school students, and it is twice as high for those who identify themselves as non-believers.

**Table 2:** Odds ratio of the first multinomial logistic regression model between Individual well-being as the dependent variable and Socio-demographic characteristics as independent variables (95% Wald Confidence Limits).

I logistic regression model				
Independent variable*	Dependent variable	Total OR	Male OR	Female OR
Class (I)	Individual well-being (positive)	1.394 (1.099–1.768)	2.002 (1.452–2.762)	/
Type of school (lyceum)	Individual well-being (negative)	1.537 (1.267–1.866)	1.64 (1.249–2.153)	1.382 (1.052–1.815)
Type of school (lyceum)	Individual well-being (positive)	0.809 (0.671–0.976)	/	/
Type of school (technical institute)	Individual well-being (positive)	0.812 (0.670–0.984)	/	0.649 (0.465–0.905)
Religious beliefs (absent)	Individual well-being (negative)	1.965 (1.664–2.320)	1.95 (1.567–2.427)	2.059 (1.594–2.659)
Religious beliefs (absent)	Individual well-being (positive)	0.747 (0.635–0.879)	/	0.602 (0.463–0.782)
Parental economic status (high)	Individual well-being (negative)	0.467 (0.337–0.647)	0.386 (0.249–0.598)	0.566 (0.346–0.925)
Parental economic status (high)	Individual well-being (positive)	1.793 (1.239–2.593)	1.96 (1.149–3.342)	/
Parental economic status (medium–high)	Individual well-being (negative)	0.471 (0.363–0.612)	0.393 (0.277–0.558)	0.576 (0.388–0.856)
Parental economic status (medium–high)	Individual well-being (positive)	1.467 (1.057–2.036)	/	/
Parental economic status (medium–low)	Individual well-being (negative)	0.566 (0.434–0.738)	0.467 (0.327–0.666)	/
Citizenship (Italian)	Individual well-being (negative)	0.678 (0.507–0.906)	0.612 (0.409–0.918)	/

*Reference value: Class (V), Type of school (vocational), Religious beliefs (present), Parental economic status (low), Citizenship (foreign).

On the other hand, this likelihood is reduced by half for individuals with a high or medium–high parental economic status. When considering the differences by sex, the probability is also halved for boys with a medium–low economic status compared to a low one and for boys with Italian citizenship. For boys, the probability of having a positive individual status is doubled if they attend class I and have a high parental economic status. For girls, being non-believers reduces this probability by half. The model did not identify any significant relationship between parental cultural status and the individual well-being of adolescents.

Table 3 displays the significant relations among the relational status variable and the social interaction variables, used as independent variables, and the individual well-being which is the dependent variable. In this case, the likelihood of having a negative individual status is reduced by half for those who have a positive relational status. The same result was obtained for those who trust institutions and their father, who are not bullying victims, who are satisfied with their body, and who feel a sense of belonging to the school community. When examining the differences by sex, among girls, the probability of having a negative individual status is halved by a high level of trust in one’s mother. On the other hand, for boys, this probability is halved by trusting their friends, achieving good school performance and, to a lesser extent, by not being cyberbullied.

**Table 3:** Odds ratio of the second and the third multinomial logistic regression models with Individual well-being as the dependent variable and Relational status and Social interaction variables as independent variables (95% Wald Confidence Limits).

Independent variable*	Dependent variable	Total OR	Male OR	Female OR
II logistic regression model				
Relational status (positive)	Individual well-being (negative)	0.416 (0.318–0.543)	0.382 (0.268–0.545)	0.465 (0.308–0.699)
III logistic regression model				
Systemic trust (high)	Individual well-being (negative)	0.527 (0.405–0.686)	0.608 (0.432–0.857)	0.460 (0.301–0.702)
Systemic trust (high)	Individual well-being (positive)	1.638 (1.288–2.084)	2.010 (1.473–2.743)	/
Trust towards father (high)	Individual well-being (positive)	/	/	2.996 (1.435–6.256)
Trust towards father (medium–high)	Individual well-being (positive)	/	/	2.176 (1.012–4.677)
Trust towards father (high)	Individual well-being (negative)	0.576 (0.404–0.822)	0.370 (0.218–0.627)	0.548 (0.336–0.893)
Trust towards mother (medium–high)	Individual well-being (negative)	0.520 (0.325–0.833)	/	0.457 (0.242–0.862)
Trust towards mother (high)	Individual well-being (negative)	0.459 (0.29–0.726)	/	0.393 (0.213–0.726)
Trust towards mother (high)	Individual well-being (positive)	/	/	5.102 (1.067–24.407)
Trust towards friends (high)	Individual well-being (positive)	/	/	2.796 (1.172–6.671)
Trust towards friends (medium–high)	Individual well-being (negative)	0.637 (0.428–0.947)	0.499 (0.265–0.942)	/
Trust towards friends (high)	Individual well-being (negative)	0.585 (0.387–0.882)	0.420 (0.220–0.800)	/
Bullying victimisation (absent)	Individual well-being (negative)	0.556 (0.458–0.675)	0.550 (0.434–0.698)	0.460 (0.323–0.655)
Bullying victimisation (absent)	Individual well-being (positive)	1.561 (1.321–1.845)	1.777 (1.428–2.211)	1.416 (1.078–1.859)
Cyberbullying victimisation (absent)	Individual well-being (negative)	0.708 (0.582–0.862)	0.585 (0.445–0.768)	/
Cyberbullying victimisation (absent)	Individual well-being (positive)	1.940 (1.461–2.577)	2.111 (1.386–3.215)	2.002 (1.341–2.990)
School connectedness (yes)	Individual well-being (negative)	0.546 (0.461–0.646)	0.507 (0.405–0.634)	0.587 (0.450–0.766)
School connectedness (yes)	Individual well-being (positive)	1.795 (1.488–2.166)	1.424 (1.122–1.808)	2.610 (1.901–3.581)
Body satisfaction (yes)	Individual well-being (negative)	0.442 (0.372–0.525)	0.373 (0.299–0.465)	0.362 (0.265–0.495)
Body satisfaction (yes)	Individual well-being (positive)	2.443 (2.027–2.944)	2.632 (1.988–3.485)	3.022 (2.305–3.962)
Interparental conflicts (absent)	Individual well-being (positive)	1.773 (1.143–2.751)	2.215 (1.136–4.318)	1.859 (1.005–3.437)
Academic performance (high)	Individual well-being (negative)	0.462 (0.341–0.626)	0.479 (0.321–0.713)	/
Academic performance (high)	Individual well-being (positive)	1.782 (1.217–2.607)	1.665 (1.047–2.646)	/
Academic performance (medium)	Individual well-being (negative)	0.543 (0.421–0.699)	0.579 (0.422–0.794)	/
Hyperconnection (absent)	Individual well-being (positive)	/	/	1.429 (1.095–1.865)

*Reference value: Social interaction (negative), Systemic trust (low), Trust towards father (low), Trust towards mother (low), Trust towards friends (low), Bullying victimisation (present), Cyberbullying victimisation (present), School connectedness (No), Body satisfaction (No), Interparental conflicts (present), Academic performance (low), Hyperconnection (present).

On the other hand, the absence of bullying victimisation, feeling a sense of belonging to the school community, and the absence of conflicts between parents increase the probability of having a positive individual well-being by more than one and a half times. Additionally, the absence of cyberbullying doubles this probability, while body satisfaction increases it by about two and a half times. When examining the differences by sex, among girls, trust towards both their father and friends triples this probability, while trust towards their mother increases it by five times and the absence of hyperconnection increases it by one and a half times. Among boys, systemic trust and a good school performance doubles the probability of having a positive individual well-being.

Table 4, on the other hand, highlights the significant association between the individual well-being, which in this case used as the independent variable, and the frequency of suicidal ideation, which was the dependent variable. Experiencing a negative individual status increases the likelihood of frequent suicidal ideation by eight times and the likelihood of experiencing suicidal ideation only once by three times. Conversely, having a positive individual well-being reduces the probability of frequent suicidal ideation by approximately 80% and the probability of experiencing suicidal ideation only once by 70%.

**Table 4:** Odds ratio of the fourth multinomial logistic regression model with Suicidal ideation as dependent variable and Individual well-being as the independent variable (95% Wald Confidence Limits).

IV logistic regression model				
Independent variable	Dependent variable	Total OR	Male OR	Female OR
Individual well-being (negative)	Suicidal ideation (more than once)	8.220 (6.746–10.015)	9.954 (7.620–13.004)	8.037 (5.775–11.183)
Individual well-being (negative)	Suicidal ideation (once)	2.967 (2.425–3.63)	3.406 (2.655–4.369)	2.664 (1.864–3.807)
Individual well-being (positive)	Suicidal ideation (more than once)	0.170 (0.121–0.239)	0.185 (0.108–0.320)	0.141 (0.091–0.218)
Individual well-being (positive)	Suicidal ideation (once)	0.312 (0.251–0.388)	0.286 (0.207–0.394)	0.304 (0.224–0.412)

All the tables presented below display odds ratios (OR) with a significant chi-square value of p < 0.05 and confidence intervals entirely above or below 1, which strengthens the reliability of the models. The modality of each variable is shown in parentheses, and the symbol “/” indicates that there were no associations between the variables.

## Discussion

**Figure 1:**
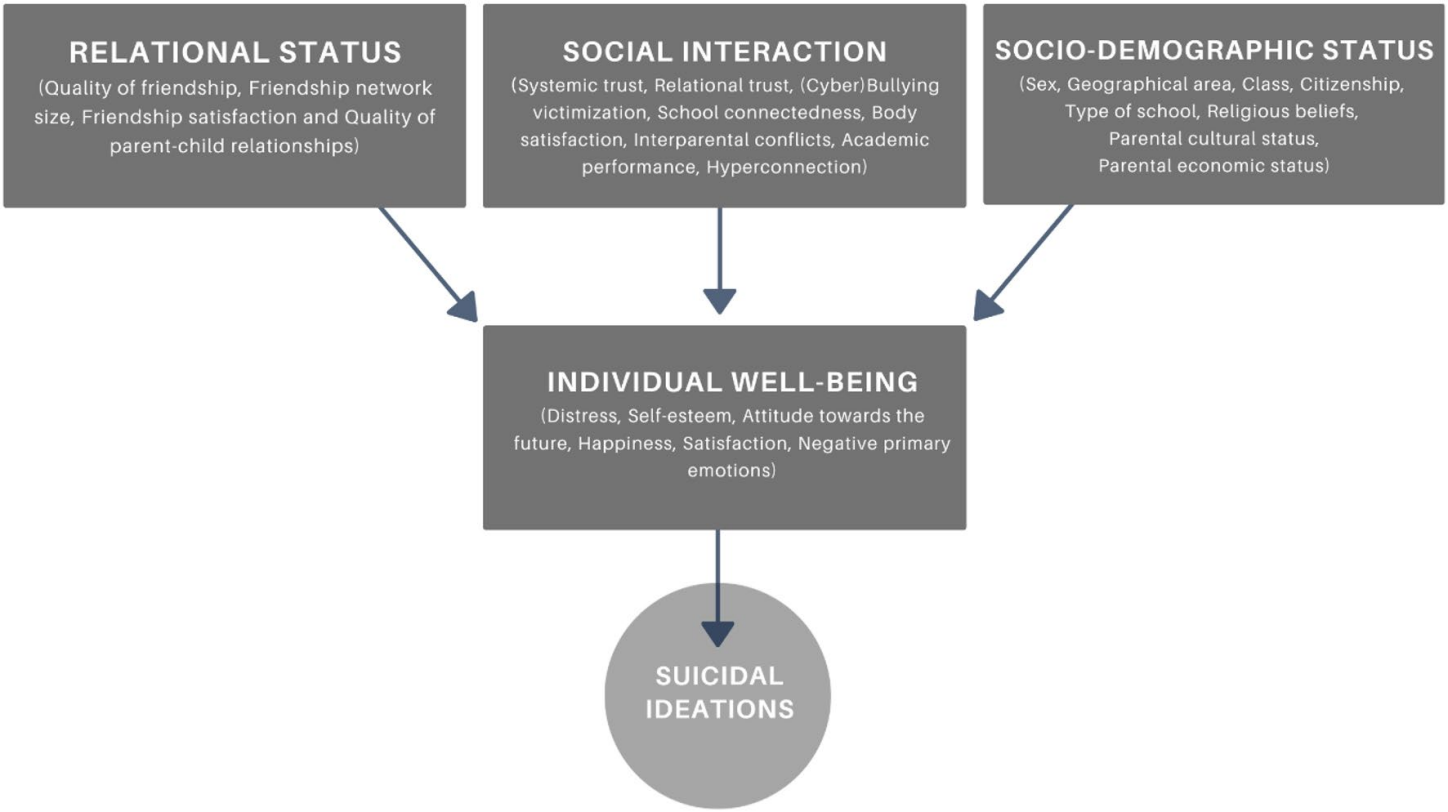
Descriptive model of the developmental process of suicidal ideation.

The results of this study shed light on demographic, social and psychological factors directly and indirectly associated with suicidal ideation among adolescents in Italy. They support our hypotheses, showing the existence of a direct association between a negative psychological status and suicidal ideation, clarifying that the psychological aspects are associated with sociodemographic characteristics of adolescents and have their origins in the social sphere. These results allowed us to build a descriptive model of development process of suicidal ideation among adolescents in Italy (Fig. 1).

Looking at the socio-demographic characteristics, the higher prevalence of suicidal ideation among female students, that aligns with previous studies^18,45^, can be explained by the fact that girls often exhibit higher levels of distress, lower self-esteem, more intense of negative emotions and greater body dissatisfaction. This gender gap in mental health is strongly influenced by social gender norms and aesthetic models^46^. Concerning the respondents’ age, the highest proportion of students experiencing frequent suicidal ideation is among those attending the second grade, aged between 14 and 15 years. This result is consistent with a study of 370,568 students where suicidal ideation was particularly common among adolescents aged between 14 and 15 years, a delicate age characterised by increased psychological instability^45^. Furthermore, in line with other studies, adolescents from lower economic backgrounds more frequently experience suicidal ideation compared to their peers with higher economic status, as poverty is considered a risk factor for worse mental health^45,47^.

The higher frequency of suicidal ideation among adolescents in the northern regions, foreign respondents, and non-believers, instead testifies the crucial role of social integration. Indeed, social interactions in Italy tends to be closer in the central-southern regions compared to the north^47,48^, while the highest risk of suicidal behaviors in adolescentes with a migration background could be linked to the challenges of acculturation but also to their disadvantaged socio-economic conditions, which similarly constitute a limit to full integration^49^. Also the protective role of religion can be attributed to factors related to active participations in religious communities where individuals often experience close and supportive relationships^50^.

The central role of social integration is shown by the multinomial logistic regression model, which examines the relationships between these sociodemographic variables and individual well-being. In this case, the absence of religious beliefs, a low economic status, and attending a lyceum are associated with a negative individual well-being, while having Italian citizenship and a higher economic status are protective factors. Contrary to our assumption, parental cultural status does not play a significant role. This finding may be due to the strong impact of the 2020 pandemic on the mental health of all adolescents, regardless of socio-demographic characteristics. However, lyceum students may be more susceptible to suicidal ideation because of formal and less frequent interactions with their parents, who, in the presence of a high cultural and professional status, may spend less time with their children and delegate their care to relatives or professionals. This would differentiate lyceum students, who are oriented towards relational models similar to those in Northern Europe, from their peers who experience more "traditional" and therefore cohesive family relationships. Additionally, lyceum students may face higher parental and social expectations, beginning with school performance.

The significant impact of social interaction on the adolescents’ individual well-being has been shown by the second multinomial logistic model. It is worth highlighting that the most influential variables in the construction of this latent factor were the size of friendships, satisfaction with friendships, the quality of relationships with friends, and the quality of parent–child relationships. The findings of this model are consistent with previous studies, which have highlighted the importance of the quality of parent–child relationships^29,30^, parenting style^51^, as well as perceived peer support and quality of friendships in the development of depressive symptoms and suicidal ideation^52^.

Looking at the sphere of attitudes and behaviours, the main factors influencing individual well-being during adolescence are related to school connectedness, academic performance, the time spent on social media, body dissatisfaction, but also the family atmosphere. Another crucial experience deserving particular attention is involvement as victims in bullying and cyberbullying. These phenomena are also strongly associated with the school environment and, as shown by both cross-sectional and longitudinal studies, they can have a detrimental impact on the mental health of adolescents, increasing the likelihood of isolation, psychological distress, and anxiety, while reducing overall well-being^53,54^.

In contrast to the results of prior studies^17^, tolerance towards alcohol and other substance use did not emerge as a significant factor in relation to the well-being of adolescents and the epistemological approach adopted in this study could account for this result. Indeed, by simultaneously controlling for the direct and indirect influence of numerous variables of both sociological and psychological nature, we were able to emphasise the most significant factors while leaving less contributing factors in the background of the phenomenon, such as the tolerance of alcohol and other psychotropic substances.

The evidence of the weight exerted by individual discomfort on suicidal ideation explains the large quantity of studies conducted worldwide considering only the psychological aspects of the phenomenon. Although the results indicate that, in comparison to their peers, a boy and a girl with compromised well-being respectively face a tenfold and eightfold higher risk of experiencing frequent suicidal ideations, analysing the phenomenon only from a psychological perspective overlooks the dynamics of social interactions, which are the primary drivers of suicidal ideation. For this reason, only an interdisciplinary approach can help us identify the sources of psychological discomfort that are always situated within social, spatial, and socio-demographic contexts.

The value of this study, in terms of advancing the understanding of suicidal ideation, lies in the interdisciplinary analysis of attitudes, behaviours, and the psychological status of Italian adolescents. The study's significance is further underscored by the wide array of sociological and psychological variables considered, the extensive and statistically representative national sample of adolescents, the use of face-to-face interviews conducted by researchers rather than external interviewers, and the inclusion of an electronic questionnaire with a design that has been tested on the same population segment for the past decade by the research group conducting this investigation. However, the study is based on a cross-sectional survey and has specific limitations. The synchronous analysis presented primarily offers a snapshot of the post-pandemic youth universe. This limitation is partially mitigated by the fact that suicidal ideation was assessed retrospectively. Nonetheless, it is advisable to validate these results through a longitudinal study that provides a diachronic view of the phenomenon. Furthermore, this study lacks qualitative insights, which are useful for a more detailed, complex, and holistic interpretation of the causes of suicidal ideation during adolescence.

In light of these considerations, we believe that the proposed descriptive model is valuable for the analysis and design of interventions to prevent suicidal ideation. Considering the serious impact of the COVID-19 pandemic on the mental health of adolescents, the consequent intensification of negative primary emotions and the shift from in-person social interactions to a virtual environment since 2020, there is an urgent need for context-specific and targeted interventions for school students. These interventions should be informed by scientific studies related to adolescents’ well-being, the risks of hyperconnection, deviance and relational violence, both in real and virtual environments, ties and trust in vertical and horizontal interaction, emotion regulation and self-esteem, as well as to gender roles and stereotypes considering the different socialization processes^55^. In this scenario, strengthening the social role of schools is essential to provide concrete support for the healthy development of young people Starting from primary school, emphasis should be placed on the school’s socialising role in promoting interaction, social integration, and actively addressing inequalities and social conditioning that are often perpetuated from one generation to the next by parents^55^. In particular, addressing issues such as hyperconnection, youth involvement in bullying and cyberbullying and the pressure of social beauty standards is of utmost importance, as these phenomena are on the rise and significantly compromise the adolescents’ well-being.

## Materials and methods

### Sample

This study is based on a national survey conducted in Italy among adolescents attending public upper secondary schools. A two-stage stratified cluster sample was used to obtain a nationally representative sample. In the first stage, the national territory was divided into 5 geographical areas (North-West, North-East, Centre, South, and Islands). For each of these areas, three large cities (with at least 100,000 inhabitants) were randomly selected, resulting in a total of 15 cities. In the second stage, three upper secondary schools were randomly selected for each city with an equivalent reserve to ensure the possibility of substitution without corrupting the sampling design, with one school representing each type according to the Italian school system: vocational institutes, technical institutes, and lyceums. The selection of schools was based on lists provided by the Italian Ministry of University and Research (MUR), resulting in a total of 45 schools. Finally, within each school, five classes were randomly selected—one for each grade—leading to a total of 225 classes. The final representative sample consisted of 4288 respondents, of whom 41.2% were female. In terms of the type of school, 38.9% of the students attended a lyceum, 31.9% a technical institute, 29.2% attended a vocational school. Regarding the school class, 21.9% of the participants were in class I, 20.6% in class II, 19.2% in class III, 19.6% in class IV, and 18.7% in class V. As for the geographical areas, 20.1% lived in the north-east, 20.4% in the north-west, 20.0% in the centre, 20.2% in the south, and 19.3% in the islands. Respondents with foreign citizenship accounted for 7.3% of the sample—this data is in line with the official statistics on the resident foreign population in Italy (Istat, 2023). The results presented in this study pertain to adolescents with the characteristics outlined in the described sample.

### Procedures and research tool

The survey was conducted between October 2021 and April 2022 using the CAPI (Computer-Assisted Personal Interviewing) method. Informed consent was obtained from all subjects and/or their legal guardian(s), and students were informed about their participation’s anonymous and voluntary nature. The study received approval from the Research Ethics and Integrity Committee of the Italian National Research Council on [approval date: 22 July 2021]. The electronic questionnaire was administered in the presence of research team members to ensure better field control and data quality. This approach allowed the respondents full autonomy in choosing their answers, better understanding of the questions, and minimized mutual interference. Furthermore, this method helped limit teachers’ interference during the data collection process, reducing the risk of bias due to their presence. The semi-structured questionnaire consisted of 77 questions covering various dimensions of analysis, including socio-economic family background, demographic information, family climate, lifestyle, leisure time activities, interaction with peers, use of social media, online behaviours and events, social deviance, stereotypes, risk behaviours, opinions, values, relational and systemic trust, individual well-being, emotions, prosociality and self-esteem. Finally, all methods in this study were carried out in accordance with relevant guidelines and regulations.

### Measures

This paragraph provides an overview of the main variables and indicators employed for the purposes of this study, along with their corresponding sample distributions indicated in parentheses.

Frequency of suicidal ideation: To assess the occurrence of suicidal ideation, the following question was presented to the participants: "Up to this point, how often have you had thoughts about suicide?". The answers were given on a 5-point scale: 'never', 'only once', 'sometimes', 'often' and 'always'. Subsequently, by summing the last three modalities, this variable was recoded into three categories: ‘never’ (55.1%), ‘only once’ (23.2%) and ‘more than once’ (21.7%). This classification allowed us to identify three groups of respondents: those who never thought about suicide, those who had suicidal ideation only once, ideally identifiable as low-risk individuals, and adolescents who, on the other hand, experienced repeated suicidal thoughts, whom we consider potentially at higher risk. Given that the phenomenon is relatively rare, the sum of the last three categories allowed us to obtain a sufficiently large group for the purposes of the analysis.

#### Measures from the group of socio-demographic variables

Parental economic status: the parental economic status was collected using two variables which detect the perception of the income of each respondent’s parents, with response modes including ‘earn a lot’, ‘earn enough’, ‘earn little’, and ‘earn nothing’. It is important to note that these variables do not represent actual income but measures the perceived economic capacity of family to which they belong. In the first stage of the recoding process, two economic status indicators were created: one for the fathers and one for the respondents’ mothers, each consisting of three levels: low, medium, and high. Subsequently, these indicators were combined in the second stage of the recoding process, simultaneously considering both parents’ responses. This resulted in an indicator of the parental economic status with four levels: low (11%), medium–low (31.8%), medium–high (44.6%), and high (12.6%).

Parental cultural status: the parental cultural status was collected using two variables which detect the educational qualification of the respondent’s parents on a five-point scale, ranging from 0 (absence of educational qualifications) to 6 (bachelor’s degree and over). In the first stage of recoding, two indicators were generated to represent the educational levels of the respondents’ fathers and mothers, respectively. These indicators were categorized into three levels of education: low, medium, and high. The second stage of recoding involved the previous indicators, combining the educational levels of both parents simultaneously. As a result, the indicator of parental cultural status was obtained, encompassing three levels: low (19.6%), medium (49.2%), and high (31.2%).

Religious beliefs: information regarding adherence to religious beliefs has been collected through a 5-point scale that measures the degree of religiosity, ranging from strong believer to indifferent. For the purposes of this research, this variable has been dichotomized, distinguishing between believers and non-believers.

#### Measures from the group of social interaction variables

Social interactions: this variable was derived through Multiple Correspondence Analysis (MCA). The multivariate method was employed to synthesise information derived from variables related to the quality of family and peer relationships as well as the size of the friendship network. The original variables identified quality of friendship, friendship network size, friendship satisfaction and quality of parent–child relationships. The result is a latent factor that primarily represents challenging relationships with peers—encompassing the friendship network size, friendship satisfaction, and quality of friendship—and, to some extent, with parents. High values of this variable correspond to significant relational difficulties. Subsequently, the obtained variable was dichotomised to identify respondents with low-quality social interactions and lacking friendships. This choice was based on the geometric shape of the variable, which showed strong polarization towards extreme values.

All the variables employed to obtain the social interactions variable are outlined below:

- Quality of peer friendships. This information was derived from a variable employing the semantic differential technique, which presented three pairs of adjectives with opposite meanings to describe one’s friends: Cold/Affectionate, Boring/Funny, False/Sincere. Each response was collected on a scale from -3, indicating closeness to the negative adjective, to 3, indicating closeness to the positive adjective, with zero denoting neutrality. The mean of the scores was then computed to gauge the quality of friendships across three levels: low (3.5%), medium (32.7%), and high (63.8%).
- Peer friendship network size. To examine the size of participants’ friendship networks, respondents were asked to specify the number of their close friends by choosing from four possible options: zero, from one to three, from four to six, and more than six. The distribution of close friends varies as follows: 50.9% of respondents reported to have one to three close friends, 31.0% four to six, 11.7% more than six, while the remaining 6.4% have no friends.
- Frequency of getting together with peers. Respondents were asked to indicate on a 4-point frequency scale how often they met with peers in their free time: ‘never’, ‘once a week’, ‘two or more times a week’ and ‘every day’: 29.1% of respondents meet their friends once a week, 46.6% two or more times a week, 14.6% every day, while 9.7% of them never meet their friends.
- Peer friendship satisfaction. To assess the perceived level of satisfaction with their peers, respondents were asked to rate their perceived satisfaction on a 4-point scale ranging from ‘not at all’ to ‘very much.’ The results showed that 44.1% were very satisfied with their friendships, 46.5% expressed a fair level of satisfaction, 7.9% were not very satisfied, while the remaining 1.5% were not satisfied at all.
- Quality of parent–child relationships. To detect the quality of the relationships between respondents and their parents, two distinct variables were employed, one regarding the relationship with fathers and another with mothers, using the semantic differential technique^45^. They present four pairs of adjectives with opposing meanings to describe the parent–child relationship: Cold/Affectionate, Conflictual/Peaceful, Authoritarian/Permissive, Morbid/Balanced. During the recoding process, each response was assigned a score ranging from − 3, indicating proximity to the negative adjective, to 3, representing proximity to the positive adjective, with zero signifying neutrality. Subsequently, the average of the obtained scores was calculated to gauge the quality of relationships on three levels: poor (mother 17.7%; father 21.1%), medium (mother 50%; father 49.2%), and high (mother 32.3%; father 29.7%).

Relational trust: to collect information about the relational trust three different variables have been employed detecting respectively the trust towards respondents’ father, mother and friends on a 4-point scale, ranging from ‘not at all’ to ‘very much’. The recoding process has resulted into three variables identifying four distinct levels of trust: high (father 58%; mother 67.9%; friends 32.2%), medium–high (father 24.6%; mother 21.5%; friends 47.4%), medium–low (father 11.1%; mother 7.5%; friends 16.5%) and low (father 6.2%; mother 3.1%; friends 3.8%).

Systemic trust: the indicator investigated trust levels towards institutions and social actors by detecting participants’ trust on a 4-point scale, ranging from ‘not at all’ to ‘very much’, towards the following entities: Scientists, Health system, School system, Police, Justice system, European Union, Banks, Journalists, Pope, Government, Catholic Church and Politicians. By calculating the mean of the obtained scores, we identified three distinct levels of systemic trust: high (24.1%), medium (49.7%), and low (26.2%).

Body satisfaction: to investigate the respondents’ perception of their own body, they were asked to indicate whether they were satisfied or dissatisfied with their bodies at the time of the interview. In our sample, 55.9% of respondents reported being satisfied with their bodies, while 44.1% expressed dissatisfaction.

Tolerance towards the use of alcohol and other substances: the indicator was created by detecting respondents’ attitudes towards the use of alcohol, marijuana, cocaine, heroin and LSD. Each tolerable use was assigned one point. After summing the scores, three levels of tolerance were identified: low (20.9%), medium (43.9%), and high (35.2%).

Bullying victimisation: the prevalence of bullying victimisation was assessed by providing respondents with a list of violent and discriminatory actions and asking if they had experienced any of these actions at their school. The list included actions such as exclusion from a group, threats, coercion, theft, physical assaults, sexual advances, and insults based on ethnicity, religious beliefs, appearance, gender, sexual orientation, academic performance, and family’s economic vulnerability. During the recoding process, a dummy variable was created to identify individuals who had suffered at least one of these acts of violence and discrimination during their time at school, amounting to 59.8% of the respondents.

Cyberbullying victimisation: the prevalence of cyberbullying victimisation was assessed by providing respondents with a list of online actions. Participants were asked to indicate the frequency of their experiences with these actions within the past year on a 4-point scale ranging from “never” to “always”. The actions included insults, teasing, threats, exclusion from groups and unauthorized sharing of personal photos or videos. After the recoding process, a dummy variable was obtained to identify individuals who had encountered at least one of these online actions with a frequency of “always” or “often”, amounting to 19.1% of the respondents.

Hyperconnection: this information has been obtained from a variable aimed at detecting the daily time spent on social media, measured in hours. Initially, collected data were categorised into four levels of screen time: absent, attributed to individuals who do not use social media (1%); low, encompassing those who spend between thirty and sixty minutes per day on social media (14.1%); medium, corresponding to usage ranging from one to three hours per day (45.4%); high, representing those who spend more than three hours daily on social media (39.4%). Subsequently, this variable was dichotomised to identify respondents with a high level of screen time (39.4%), deemed hyperconnected, while 0 indicating the absence of hyperconnection.

#### Measures from the group of the psychological variables

Individual well-being: this variable, obtained from the application of Multiple Correspondence Analysis (MCA), integrates information from six variables and indicators detecting different psychological aspects: self-esteem, psychological distress, intensity of negative primary emotions, happiness, satisfaction, and attitudes towards the future. The result is a latent factor representing positive individual well-being, primarily characterised by high levels of self-esteem, happiness and satisfaction, the absence of distress, and a low intensity of negative emotions. Subsequently, the obtained variable was categorized by reducing it into quartiles and selecting the first and third quartiles to create a three-category variable. This categorization allowed us to identify respondents with a high positive individual well-being and those with a high negative individual well-being. This choice was made to highlight the tails of the distribution, which are the most significant categories for the analysis of the phenomenon. All the measures employed in creating the Individual well-being variable are described below:

- Self-esteem. The Rosenberg self-esteem scale, a well-established instrument in social science research^56^, was employed to detect the level of respondents’ self-esteem, defined as a positive or negative attitude towards oneself. This indicator is divided into low (34.1%), healthy (49.3%) and high (16.7%) self-esteem and it is non-linear because the ‘desirable’ scores are distributed in the middle of the scale.
- Psychological distress. To detect anxiety and depressive symptoms, we used the Kessler scale of psychological distress (K10)^57^. This scale consists of 10 questions, with responses graded into five levels, measuring the frequency of perceived anxious-depressive symptoms such as nervousness, sadness, restlessness, hopelessness and feelings of worthlessness. The sum of the scores, ranging from 10 to 50, defined four levels of psychological distress: absent (31.9%), low (21.5%), moderate (17.1%) and high (29.5%).
- Intensity of negative primary emotions. The intensity of negative primary emotions was collected on a seven-point response scale, with 1 indicating the lowest intensity perceived and 7 the highest intensity, with reference to the following three negative emotions: anger, sadness and fear. The mean of the scores have been calculated to create a composite indicator, which was then used to categorise participants into three levels of intensity for negative primary emotions: high (30%), medium (40.6%) and low (29.4%).
- Happiness and Satisfaction. The two variables detect the perception of happiness (H) and satisfaction (S) of the respondents using a Cantril scale ranging from 1 (representing the lowest level) to 10 (indicating the maximum level). During the recoding procedure, scores from 1 to 6 were grouped, as were scores from 7 to 8 and those from 9 to 10. In this way three distinct levels of happiness and satisfaction were obtained: low (H 32%; S 27.9%), medium (H 32.4%; S 37.1%) and high (H 35.6%; S 35%).
- Attitudes towards the future. The negative perceptions regarding the future relied on two distinct indicators which detect respectively pessimism and uncertainty about forthcoming events. Both aspects were operationalised through the following two statements “I hold a positive outlook on the future” and “My future appears uncertain” using a 4-point Likert scale. The final index is a binary variable where 1 denotes respondents exhibiting negative perceptions of the future and a sense of uncertainty (30.9%), while 0 encompasses all other cases (69.1%).

### Data analysis

The analysis of data was carried out using two different software: SPSS (version 28, IBM, Chicago, IL, USA) and SAS (version 9.4, © 2023 SAS Institute Inc.). The analysis employed various methods. Initially, a bivariate analysis was performed to observe the variations in suicidal ideation in relation to specific socio-demographic variables (Table 1). Furthermore, a bivariate analysis between all the variables of the study and suicidal ideation has been carried out (Appendix 1). Subsequently, multivariate approaches were employed for more in-depth analysis. After conducting a comprehensive analysis of the data distribution, which included cross-tabulations of the variables of interest, we evaluated a multi-mode model involving concatenating multinomial logistic models. This particular model adopts a path analysis structure (Fig. 1). Furthermore, in order to synthesise peer interactions and individual well-being measures and to create a more robust and dependable measurement tool, we employed the multivariate method known as Multiple Correspondence Analysis (MCA). This goal was successfully achieved, as the MCA analysis revealed that these variables accounted for 79% and 93% of the explained variance, respectively. Subsequently, we categorised these variables and utilized them as independent and dependent variables in logistic models.

The logistic regression model tests the null hypothesis (H0), which assumes statistical independence between the variable y (the target variable) and x (the effects). In this hypothesis, H0: β = 0, meaning that x has no influence on the success probability P (y = 1). To test H0, we use the Z-test, which involves dividing the maximum likelihood estimate of β by its standard error. This ratio is referred to as the Wald statistic and follows a chi-square distribution. For nominal response variables, an extension of the logistic regression model provides a logistic model for each pair of response categories. In our application, a reference category was selected and compared to every other category. In these models, it is assumed that the observations are independent and follow a multinomial distribution. To select significant variables, we employ the forward procedure. Step by step, the procedure calculates the score chi-square statistic for each effect not in the model and evaluates the largest of these statistics. If it is significant (at the 0.05 level), the corresponding effect is added to the model. Once an effect is included in the model, it is retained and not removed. The process is repeated until none of the remaining effects meets the specified entry level. For each selected effect, we analysed the maximum likelihood estimates of the parameters and assessed their significance.

**Figure 2:**
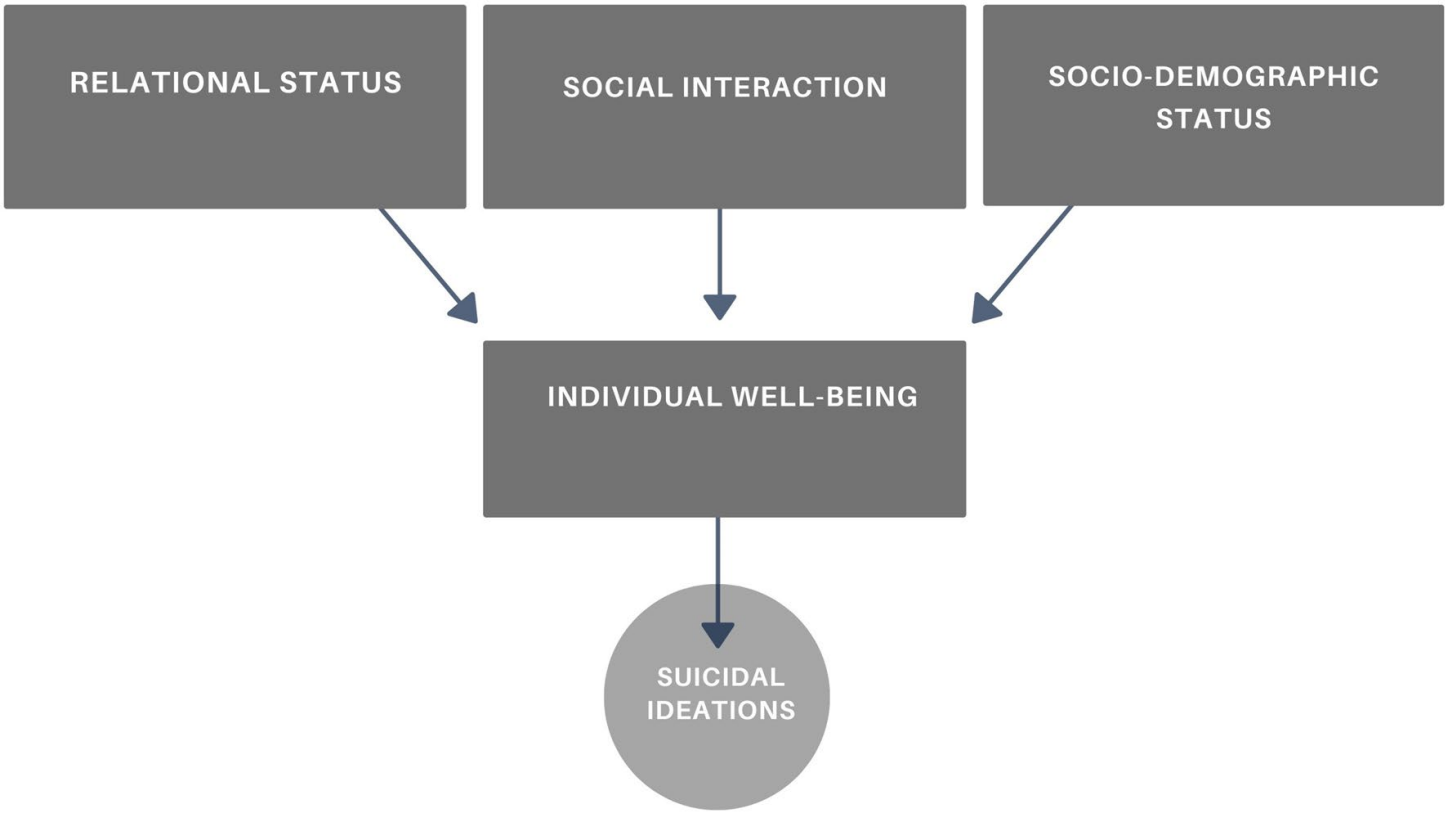
Path analysis structure of the multinomial logistic model.

Figure 2 illustrates the theoretical model’s structure, outlining the hypothesised relationships among variables. Individual well-being is hypothesised to be influenced by the respondents’ social environment, behaviours and certain socio-demographic characteristics, affecting suicidal ideation. Given that individual well-being directly impacts the presence and frequency of suicidal ideations, the latter are believed to be indirectly influenced by specific factors related to the social environment and certain socio-demographic characteristics.

To support this hypothesis, multinomial logistic regression models that included the variables of the relational status group, the social interaction factor, and sociodemographic variables as independent variables, with suicidal ideation as the dependent variable were first carried out. Subsequently, the same models were replicated, incorporating the latent factor of individual well-being among the independent variables. In this second models, the influence of the other independent variables was found to be absorbed by individual well-being (Appendix 2).

### Supplementary Information

Supplementary Information 1.

Supplementary Information 2.

## Data Availability

The data that support the findings of this study are available from the corresponding author upon reasonable request.
