# Supplementary material for: The developmental process of suicidal ideation among adolescents: social and psychological impact from a nation-wide survey
Source: Sci Rep. 2023 Nov 28;13:20984. doi: 10.1038/s41598-023-48201-6 (PMC10684516; doi:10.1038/s41598-023-48201-6)
Supplement: Supplementary file 1 — Supplementary Information 1. [file 41598_2023_48201_MOESM1_ESM.pdf]

Appendix 1

Bivariate relationships between the frequency of suicidal ideation and the variables of the following groups: relational status, social interaction, and individual well-being.

Table 1. Frequency of suicidal ideation by relational status group's variables (n)

		More than once	Once	Never
**Quality of friendship**	Low	32.3% (46)	23.9% (34)	43.6% (62)
	Medium	23.5% (309)	24.5% (322)	51.9% (582)
	High	18.5% (475)	22.7% (582)	58.7% (1503)
**Quality of father-child relationships**	Low	35.7% (315)	26.0% (229)	38.1% (336)
	Medium	20.1% (413)	24.3% (500)	55.5% (1140)
	High	13.9% (173)	18.6% (232)	67.3% 837)
**Quality of mother-child relationships**	Low	35.7% (270)	27.3% (207)	36.9% (279)
	Medium	21.7% (464)	24.2% (517)	54.0% (1155)
	High	13.6% (189)	19.3% (267)	66.9% (924)
**Friendship satisfaction**	Not at all	51.5% (33)	14.0% (9)	34.3% (22)
	Slightly	38.2% (129)	28.7% (97)	32.9% (111)
	Moderately	21.6% (432)	25.5% (510)	52.8% (1055)
	Very much	17.7% (336)	20.0% (378)	62.2% (1176)
**Frequency of getting together with peers**	Never	31.1% (129)	21.2% (88)	47.6% (197)
	Once a week	27.9% (349)	25.8% (322)	46.3% (579)
	Two or more times a week	17.6% (353)	23.6% (472)	58.7% (1175)
	Every day	15.9% (99)	17.9% (112)	66.2% (413)
**Friendship network size**	None	36.4% (100)	20.4% (56)	43.0% (118)
	From 1 to 3	23.0% (503)	24.7% (541)	52.1% (1140)
	From 4 to 6	19.2% (256)	22.4% (229)	58.2% (775)
	6>	14.2% (71)	19.6% (98)	66.2% (331)
**Relational status (latent factor)**	Negative	37.4% (107)	20.3% (58)	42.3% (121)
	Positive	20.6% (823)	23.4% (936	56% (2243)

Table 2. Frequencies of suicidal ideation by social interaction group's variables (n)

		More than once	Once	Never
**Systemic trust**	Low	30.2% (339)	23.5% (264)	46.2% (519)
	Medium	20.4% (435)	25.1% (535)	54.5% (1162)
	High	15.1% (156)	18.8% (195)	66% (683)
**Trust towards father**	Absent	47.7% (124)	23.8% (62)	28.4% (74)
	Low	40.3% (187)	28.% (130)	31.7% (147)
	Medium	27.9% (287)	26.7% (274)	45.4% (466)

	High	12.5% (303)	20.4% (495)	67.1% (1625)
**Trust towards mother**	Absent	55.3% (73)	20.4% (27)	24.2% (132))
	Low	47.3% (152)	27.7% (89)	24.9% (321)
	Medium	30.3% (279)	27.9% (257)	41.7% (920)
	High	14.5% (422)	21.3% (618)	64.2% (2904)
**Trust towards friends**	Absent	35.6% (58)	23.9% (39)	40.5% (66)
	Low	32.3% (229)	24.4% (173)	43.3% (307)
	Medium	18.8% (382)	24.6% (500)	56.6% (1152)
	High	18.9% (261)	20.4% (282)	60.7% (839)
**Cyberbullying victimisation**	Absent	16.8% (583)	22.6% (786)	60.5% (2098)
	Present	42.2% (347)	25.3% (208)	32.4 % (266)
**Bullying victimisation**	Absent	11.9% (205)	15.4% (265)	72.7% (1255)
	Present	28.3 % (725)	28.4% (729)	43.3% (1109)
**Hyperconnection**	Absent	18.7% (485)	23.1% (601)	58.2% (1511)
	Present	26.3% (445)	23.2% (393)	50.4% (853)
**School connectedness**	Absent	30% (506)	25.1% (424)	44.9% (757)
	Present	16.3% (424)	21.9% (570)	61.8% (1607)
**Tolerance towards alcohol and other substances**	High	33.1% (296)	24.1% (216)	42.8% (383)
	Medium	22.1% (415)	25.5% (481)	52.4% (988)
	Low	14.5% (219)	19.7% (297)	65.8 % (993)
**Body satisfaction**	Present	13.4% (322)	20.1% (481)	66.4% (1593)
	Absent	32.1% (608)	27.1% (513)	40.7% (771)
**Academic performance**	High	18.1% (171)	23.1% (218)	58.8% (556)
	Medium	21.2% (619)	23.2% (680)	55.5% (1625)
	Low	33.4% (140)	22.9% (96)	43.6% (183)
**Interparental conflicts**	Present	32.5% (420)	26.7% (346)	40.7% (527)
	Absent	16.5% (474)	21.3% (612)	62.1% (1780)

Table 3. Frequencies of suicidal ideation by Individual well-being group's variables (n)

		**More than once**	**Once**	**Never**
**Self-esteem**	Low	43.4% (634)	29.1% (425)	27.5% (402)
	Medium	12.6% (266)	22.9% (485)	64.4% (1362)
	High	4.2% (30)	11.8% (84)	84% (600)
**Intensity of negative primary emotions**	Low	8.9% (112)	14.7% (185)	76.4% (964)
	Medium	19.4% (338)	26.3% (458)	54.3% (946)
	High	37.3% (480)	27.3% (351)	35.3% (454)
**Attitude towards future**	Positive	14.3% (423)	22 % (650)	63.7% (1879)
	Negative	38.3% (505)	25.8% (341)	35.9% (475)

**Distress**	Absent	4.6% (63)	12.6% (172)	82.8% (1133)
	Low	10.6% (98)	25.8% (238)	63.5% (585)
	Medium	20.7% (152)	31.6% (232)	47.7% (351)
	High	48.8% (617)	27.8% (352)	23.3% (295)
**Happiness**	Low	43.1% (592)	24.8% (341)	32.1% (441)
	Medium	14.6% (203)	29% (403)	56.4% (782)
	High	8.8% (135)	16.4% (250)	74.8% (1141)
**Satisfaction**	Low	41.2% (493)	26.1% (313)	32.7% (391)
	Medium	17.8% (283)	26% (412)	56.2% (895)
	High	10.2% (154)	17.9% (269)	71.8% (1078)
**Individual well-being (latent factor)**	Low	50.2% (529)	28.4% (299)	21.4% (225)
	Medium	16.3% (360)	25.9% (573)	57.8% (1277)
	High	4% (41)	11.9% (122)	84.1% (862)
