# Supplementary material for: The developmental process of suicidal ideation among adolescents: social and psychological impact from a nation-wide survey
Source: Sci Rep. 2023 Nov 28;13:20984. doi: 10.1038/s41598-023-48201-6 (PMC10684516; doi:10.1038/s41598-023-48201-6)
Supplement: Supplementary file 2 — Supplementary Information 2. [file 41598_2023_48201_MOESM2_ESM.pdf]

Appendix 2

Table 1.1 Odds Ratio of the multinomial logistic regression models with Suicidal ideation as dependent variable and Socio-demographic characteristics as independent variables (95% Wald Confidence Limits)

**Independent variable***	**Dependent variable**	**Total OR**	**Male OR**	**Female OR**
Type of school (Lyceum)	Suicidal ideation (Once)	/	1.388 (1.05-1.833)	/
Type of school (Technical)	Suicidal ideation (More than once)	0.747 (0.608-0.917)	/	/
Cohabitating parents (Yes)	Suicidal ideation (More than once)	0.793 (0.657-0.957)	/	/
Religious beliefs (absent)	Suicidal ideation (More than once)	1.978 (1.666-2.348)	2.262 (1.761-2.905)	2.308 (1.802-2.958)
Religious beliefs (absent)	Suicidal ideation (Once)	1.265 (1.076-1.488)	1.294 (1.045-1.603)	1.513 (1.174-1.949)
Parental economic status (High)	Suicidal ideation (More than once)	0.47 (0.333-0.664)	0.514 (0.315–0.84)	/
Parental economic status (High)	Suicidal ideation (Once)	0.643 (0.463-0.893)	0.628 (0.404–0.977)	/
Parental economic status (Medium-high)	Suicidal ideation (More than once)	0.482 (0.363-0.893)	0.423 (0.282–0.636)	/
Parental economic status (Medium-high)	Suicidal ideation (Once)	0.562 (0.425-0.743)	0.531 (0.365–0.771)	/
Parental economic status (Medium-low)	Suicidal ideation (More than once)	0.64 (0.48-0.853)	0.577 (0.392–0.849)	/
Parental economic status (Medium-low)	Suicidal ideation (Once)	0.631 (0.474-0.84)	/	/
Citizenship (Italian)	Suicidal ideation (More than once)	0.542 (0.404-0.726)	0.513 (0.334–0.789)	/
Geographical area (North)	Suicidal ideation (More than once)	0.8 (0.668-0.957)	/	0.642 (0.496–0.831)
Geographical area (North)	Suicidal ideation (Once)	0.779 (0.656-0.926)	/	/

**Reference value:* Cohabitating parents (Not), *Type of school (vocational), Religious beliefs (present), Parental economic status (low), Citizenship (foreign).*

Table 1.2 Odds Ratio of the multinomial logistic regression models with Suicidal ideation as dependent variable and Socio-demographic characteristics and Individual well-being as independent variables (95% Wald Confidence Limits)

**Independent variable***	**Dependent variable**	**Total OR**	**Male OR**	**Female OR**
Religious beliefs (absent)	Suicidal ideation (More than once)	1.444 (1.193-1.748)	1.659 (1.257-2.189)	1.626 (1.227-2.154)
Type of school (Technical)	Suicidal ideation (More than once)	0.61 (0.485-0.766)	/	/
Type of school (Technical)	Suicidal ideation (Once)	0.808 (0.659-0.99)	/	/
Citizenship (Italian)	Suicidal ideation (More than once)	0.544 (0.392-0.755)	0.549 (0.339–0.889)	/
Individual well-being (negative)	Suicidal ideation (More than once)	7.961 (6.515-9.727)	9.399 (7.181-12.302)	7.588 (5.442-10.581)
Individual well-being (negative)	Suicidal ideation (Once)	2.939 (2.398-3.601)	3.363 (2.618-4.32)	2.583 (1.804-3.698)

Individual well-being (positive)	Suicidal ideation (More than once)	0.17 (0.121-0.239)	0.187 (0.108-0.322)	0.147 (0.094-0.228)
Individual well-being (positive)	Suicidal ideation (Once)	0.311 (0.25-0.387)	0.286 (0.207-0.395)	0.309 (0.228-0.42)

**Reference value: Religious beliefs (present), Citizenship (foreign), Individual well-being (Medium)*

Table 2.1 Odds Ratio of the multinomial logistic regression models with Suicidal ideation as dependent variable and Relational status as independent variables (95% Wald Confidence Limits)

**Independent variable***	**Dependent variable**	**Total OR**	**Male OR**	**Female OR**
Relational status (Positive)	Suicidal ideation (More than once)	0.411 (0.312-0.543)	0.432 (0.296-0.63)	0.37 (0.24-0.572)

**Reference value: Relational status (negative)*

Table 2.2 Odds Ratio of the multinomial logistic regression models with Suicidal ideation as dependent variable and Relational status and Individual well-being as independent variables (95% Wald Confidence Limits)

**Independent variable***	**Dependent variable**	**Total OR**	**Male OR**	**Female OR**
Individual well-being (positive)	Suicidal ideation (More than once)	0.626 (0.454-0.863)	/	0.492 (0.297-0.816)
Individual well-being (negative)	Suicidal ideation (More than once)	8.003 (6.564-9.759)	9.954 (7.62-13-004)	7.812 (5.608-10.881)
Individual well-being (negative)	Suicidal ideation (Once)	2.976 (2.431-3.644)	3.406 (2.655-4.369)	2.644 (1.849-3.781)
Individual well-being (positive)	Suicidal ideation (More than once)	0.171 (0.122-0.24)	0.185 (0.108-0.32)	0.141 (0.091-0.22)
Individual well-being (positive)	Suicidal ideation (Once)	0.312 (0.251-0.388)	0.286 (0.207-0.394)	0.304 (0.224-0.412)

**Reference value: Individual well-being (Medium)*

Table 3.1. Odds Ratio of the multinomial logistic regression models with Suicidal ideation as dependent variable and Social interaction variables as independent variables (95% Wald Confidence Limits)

**Independent variable***	**Dependent variable**	**Total OR**	**Male OR**	**Female OR**
Systemic trust (High)	Suicidal ideation (More than once)	0.653 (0.502-0.848)	/	0.402 (0.269-0.601)
Systemic trust (High)	Suicidal ideation (Once)	0.759 (0.598-0.963)	/	0.506 (0.349-0.733)
Systemic trust (Medium)	Suicidal ideation (More than once)	0.789 (0.642-0.969)	/	0.615 (0.446-0.846)
Systemic trust (Medium)	Suicidal ideation (Once)	/	/	0.72 (0.528-0.983)
Trust towards father (High)	Suicidal ideation (More than once)	0.349 (0.235-0.517)	0.264 (0.149-0.468)	/
Trust towards father (High)	Suicidal ideation (Once)	0.645 (0.43-0.968)	/	/
Trust towards mother (High)	Suicidal ideation (More than once)	0.433 (0.259-0.724)	/	0.199 (0.095-0.418)

Trust towards mother (Medium-high)	Suicidal ideation (More than once)	/	/	0.464 (0.217-0.994)
Bullying victimisation (Absent)	Suicidal ideation (More than once)	0.436 (0.357-0.533)	0.516 (0.399-0.668)	0.353 (0.255-0.487)
Bullying victimisation (Absent)	Suicidal ideation (Once)	0.414 (0.348-0.493)	0.393 (0.315-0.492)	0.44 (0.333-0.582)
Cyberbullying victimisation (Absent)	Suicidal ideation (More than once)	0.374 (0.303-0.463)	0.37 (0.276-0.497)	0.367 (0.269-0.502)
Cyberbullying victimisation (Absent)	Suicidal ideation (Once)	0.713 (0.574-0.884)	0.687 (0.513-0.92)	/
School connectedness (Yes)	Suicidal ideation (More than once)	0.604 (0.504-0.724)	0.52 (0.408-0.662)	0.664 (0.505-0.874)
School connectedness (Yes)	Suicidal ideation (Once)	0.802 (0.679-0.948)	0.706 (0.571-0.874)	/
Body satisfaction (Yes)	Suicidal ideation (More than once)	0.427 (0.357-0.512)	0.476 (0.372-0.608)	0.428 (0.323-0.568)
Body satisfaction (Yes)	Suicidal ideation (Once)	0.614 (0.522-0.723)	0.597 (0.48-0.743)	0.666 (0.516-0.858)
Interparental conflicts (Medium high)	Suicidal ideation (More than once)	0.706 (0.499-0.999)	/	0.593 (0.367-0.957)
Interparental conflicts (Absent)	Suicidal ideation (More than once)	0.441 (0.298-0.653)	/	0.263 (0.148-0.467)
Interparental conflicts (Absent)	Suicidal ideation (Once)	0.462 (0.317-0.674)	/	0.314 (0.181-0.542)
Interparental conflicts (Low)	Suicidal ideation (More than once)	0.544 (0.388-0.762)	/	0.375 (0.237-0.594)
Interparental conflicts (Low)	Suicidal ideation (Once)	0.636 (0456-0.886)	/	0.485 (0.308-0.764)

**Reference value: Systemic trust (low), Trust towards father (low), Trust towards mother (low), Bullying victimisation (present), Cyberbullying victimisation (present), School connectedness (No), Body satisfaction (No), Interparental conflicts (present).*

Table 3.2 Odds Ratio of the multinomial logistic regression models with Suicidal ideation as dependent variable and Social interaction variables and Individual well-being as independent variables (95% Wald Confidence Limits)

**Independent variable***	**Dependent variable**	**Total OR**	**Male OR**	**Female OR**
Systemic trust (High)	Suicidal ideation (More than once)	/	/	0.483 (0.318-0.733)
Systemic trust (High)	Suicidal ideation (Once)	/	/	0.557 (0.383-0.81)
Systemic trust (Medium)	Suicidal ideation (More than once)	/	/	0.627 (0.448-0.877)
Systemic trust (Medium)	Suicidal ideation (Once)	/	/	0.724 (0.528-0.992)
Trust towards father (High)	Suicidal ideation (More than once)	0.409 (0.271-0.618)	0.321 (0.185-0.559)	/
Trust towards mother (High)	Suicidal ideation (More than once)	/	/	0.337 (0.157-0.725)
Bullying victimisation (Absent)	Suicidal ideation (More than once)	0.528 (0.428-0.651)	0.644 (0.49-0.848)	0.419 (0.3-0.585)
Bullying victimisation (Absent)	Suicidal ideation (Once)	0.457 (0.383-0.545)	0.44 (0.351-0.552)	0.471 (0.355-0.625)
Cyberbullying victimisation (Absent)	Suicidal ideation (More than once)	0.43 (0.344-0.538)	0.468 (0.342-0.641)	0.407 (0.294-0.565)
Cyberbullying victimisation (Absent)	Suicidal ideation (Once)	0.798 (0.64-0.995)	/	/
Body satisfaction (Yes)	Suicidal ideation (More than once)	0.616 (0.508-0.746)	/	/

Body satisfaction (Yes)	Suicidal ideation (Once)	0.75 (0.633-0.888)	/	/
Interparental conflicts (Absent)	Suicidal ideation (More than once)	0.489 (0.323-0.739)	/	0.326 (0.178-0.597)
Interparental conflicts (Absent)	Suicidal ideation (Once)	0.497 (0.339-0.731)	/	0.357 (0.204-0.624)
Interparental conflicts (Low)	Suicidal ideation (More than once)	0.564 (0.395-0.805)	/	0.427 (0.263-0.694)
Interparental conflicts (Low)	Suicidal ideation (Once)	0.648 (0.462-0.911)	/	0.524 (0.33-0.834)
Individual well-being (negative)	Suicidal ideation (More than once)	/	7 (5.291-9.26)	5.187 (3.631-7.41)
Individual well-being (negative)	Suicidal ideation (Once)	2.266 (1.833-2.801)	2.735 (2.109-3.547)	2.049 (1.413-2.97)
Individual well-being (positive)	Suicidal ideation (More than once)	/	0.24 (0.138-0.416)	0.236 (0.149-0.373)
Individual well-being (positive)	Suicidal ideation (Once)	/	0.343 (0.247-0.477)	0.405 (0.295-0.557)

**Reference value: Systemic trust (low), Trust towards father (low), Trust towards mother (low), Bullying victimisation (present), Cyberbullying victimisation (present), School connectedness (No), Body satisfaction (No), Interparental conflicts (present), Individual well-being (Medium).*
